# The codification of hazard and its impact on the hazard versus risk controversy

**DOI:** 10.1007/s00204-021-03145-6

**Published:** 2021-09-24

**Authors:** John E. Doe, Alan R. Boobis, Samuel M. Cohen, Vicki L. Dellarco, Penelope A. Fenner-Crisp, Angelo Moretto, Timothy P. Pastoor, Rita S. Schoeny, Jennifer G. Seed, Douglas C. Wolf

**Affiliations:** 1 School of Pharmacy and Biomolecular Sciences, Liverpool John Moores University, Byrom Street, Liverpool, L3 3AF UK; 2 National Heart & Lung Institute, Hammersmith Campus, Imperial College London, London, W12 0NN UK; 3 Department of Pathology and Microbiology, Havlik-Wall Professor of Oncology, University of Nebraska Medical Center, Omaha, NE 68198-3135 USA; 4 Independent Consultant, Silver Spring, MD 20901 USA; 5 Independent Consultant, North Garden, VA 22959 USA; 6 Dipartimento di Scienze Cardio-Toraco-Vascolari e Sanità Pubblica, (Department of Cardio-Thoraco-Vascular and Public Health Sciences), Università degli Studi di Padova, Padua, Italy; 7 Pastoor Science Communication, LLC, Greensboro, NC 27455 USA; 8 Rita Schoeny LLC, Washington, DC 20002 USA; 9 Independent Consultant, Alexandria, VA 22301 USA; 10 Syngenta Crop Protection LLC, Greensboro, NC 27419 USA

## Abstract

The long running controversy about the relative merits of hazard-based versus risk-based approaches has been investigated. There are three levels of hazard codification: level 1 divides chemicals into dichotomous bands of hazardous and non-hazardous; level 2 divides chemicals into bands of hazard based on severity and/or potency; and level 3 places each chemical on a continuum of hazard based on severity and/or potency. Any system which imposes compartments onto a continuum will give rise to issues at the boundaries, especially with only two compartments. Level 1 schemes are only justifiable if there is no variation in severity, or potency or if there is no threshold. This is the assumption implicit in GHS/EU classification for carcinogenicity, reproductive toxicity and mutagenicity. However, this assumption has been challenged. Codification level 2 hazard assessments offer a range of choices and reduce the built-in conflict inherent in the level 1 process. Level 3 assessments allow a full range of choices between the extremes and reduce the built-in conflict even more. The underlying reason for the controversy between hazard and risk is the use of level 1 hazard codification schemes in situations where there are ranges of severity and potency which require the use of level 2 or level 3 hazard codification. There is not a major difference between level 2 and level 3 codification, and they can both be used to select appropriate risk management options. Existing level 1 codification schemes should be reviewed and developed into level 2 schemes where appropriate.

## Introduction

There has been a long running controversy about the relative merits of hazard-based versus risk-based approaches in managing the potential for harm to human health from the use of chemicals. Lofsted (2011) in his article ‘Risk versus Hazard—How to Regulate in the 21st Century’ outlined the issues involved. These included inconsistency in approach where countries use hazard-based legislation to ban chemicals of no consequence to their economy and risk-based legislation to keep the chemicals that matter to them. This can result in inappropriate levels of concern, either too much or too little, over some chemicals due to factors such as perception of no choice in exposure, poorly understood technical issues such as dose response, unfamiliarity with uses and benefits, and political desire to ban or to keep in use. The resultant controversy was expressed in the question “Should regulations be based on a hazard classification (that is the potential for a substance, activity or process to cause harm or adverse effect) or a risk (a combination of the likelihood and the severity of a substance, activity or process to cause harm) assessment?” (Lofsted 2011). Recently, the German Federal Institute for Risk Assessment (BfR) (Herzler et al. 2021) expressed concerns over the EU chemical strategy, including the intention to move away from a risk-based to a hazard-based assessment paradigm. They said that “such a move, tempting as it might be, is bound to create a range of problems and will likely result in a system that by design would be inherently arbitrary and inconsistent”.

In a previous paper (Boobis et al. 2016), we highlighted the issues arising from classification for cancer by hazard identification rather than by hazard characterization which results in chemicals in the same category with up to seven orders of magnitude difference in potency that causes confusion and makes it difficult to make sound decisions on risk management. In this paper, we explore how the complexities of the output from toxicological studies are simplified and codified before being used in regulatory schemes not only for cancer but for other areas such as reproductive toxicity, endocrine disruption, and mutagenicity. We conclude that the risk versus hazard controversy is fueled by the use of over simplistic binary classification schemes and that there are ways forward within the existing schemes which could avoid this problem.

## Hazard codification and the regulation of chemicals

The aim of chemical regulation is to minimize the risk of ill health caused by exposure to chemicals. To do this, the capability of a chemical to cause harm has to be known and compared with the measured or estimated human exposure. Whether a hazard-based system or a risk-based system is used, the hazard must be understood sufficiently to allow a choice among risk management options aimed at minimizing adverse effects on public health. This understanding must be both qualitative—what could it do—and quantitative—how much is needed to do it.

The term "hazardous chemical” is widely used in the EU chemical safety approach, with the policy aim to avoid exposures to "hazardous chemicals” and to replace them with less hazardous ones. A European Parliament resolution (EU 2020a) states that “the chemical, physical and toxicological properties of chemicals vary greatly” and that “a transition is needed towards producing chemicals that are safe by design, including using less hazardous chemicals”. This policy opens up the question of what is a ‘hazardous chemical’, and how would it differ from a ‘less hazardous chemical’.

There are two aspects to hazard: the adverse effects the chemical has the potential to cause; and the circumstances (including dose level, duration and route of exposure) in which it might cause them. These components of the degree of hazard are generally known as severity and potency. Clearly, there is a difference in severity between a mild transient effect such as skin reddening and a life-threatening progressive disease such as cancer. Equally, there are differences in potency between chemicals showing effects only at high doses such as grams/kg and those which cause effects at low doses such as µg/kg. There is a continuum of potency and a continuum of severity, albeit potency is easier to quantify than severity.

The GHS scheme takes into account a third aspect of the toxicity of a chemical in determining its hazard classification, that of mode of action (MOA) (United Nations 2019). The effects of a chemical at high doses may be dependent on an MOA different from that occurring at exposures even remotely possible in human populations, or it may be that the MOA for an effect is not relevant to humans due to fundamental qualitative differences in biology. In such cases, GHS takes this into account in a decision on classification, although this is not carried through into the EU scheme based on GHS.

If hazard is defined as the potential to cause harm and the toxicological properties of chemicals vary greatly, then each chemical has a combination of severity and potency which determine how hazardous it is. Thus, it is important to be able to quantify the ‘degree of hazard’ (a term used in EU Classification, Packaging and Labelling guidance, ECHA 2017) which a chemical may pose, so that appropriate risk management measures can be taken.

A full toxicological evaluation of a chemical generates thousands of pieces of information which detail the type of effects and the doses at which they occur. This information must be assessed for quality and reliability, as well as summarised and codified before it can be used for safety decision-making. We put forward the concept that there are three main levels of codification at which this is done (Table 1):

**Table 1:** Codification of hazard scheme

Hazard codification level	Summary	Risk management options
Level 1	Presence or absence of a class of adverse effect Yes or no binary choice (limited banding)	Restricts risk management and assumes all chemicals have the same severity/potency No flexibility
Level 2	Compartmentalization of hazard first by nature of adverse effect and then by potency by banding with several categories (typically 3–5) Semi-quantitative	Provides more risk management choices that reflect differences in severity/potency Some flexibility Useful when data are limited
Level 3	Description of nature of adverse effect and the derivation of a health based guidance value (e.g., acceptable daily intake, reference dose, derived no effect level) to establish safe levels of exposure Infinite bands, continuous response based on quantitative dose response risk assessment	Broad range of risk management options Maximizes flexibility and accuracy for a more reliable fit for purpose assessment

Codification levels 1 and 2 are used in classification schemes and level 3 is used in risk assessment schemes. Level 1 is binary and uses a weight of evidence approach to determine if a chemical has the potential to cause a particular class of adverse effect, although, in practice, strength of evidence is frequently used. If the chemical is considered to have the potential to cause an effect, then it is deemed to be ‘hazardous’ and if it is considered not to have the potential it is deemed to be ‘not hazardous’. Within this type of scheme, all the chemicals deemed to be hazardous are considered as if they have the same severity and potency of effect. Level 1 schemes are applied to carcinogenicity, mutagenicity, and reproductive toxicity (CMR) within the EU/GHS scheme (ECHA 2017). Risk management decisions are then taken based on the assumption of the same severity and potency. The risk management decisions also tend to be binary, i.e., ban or allow over broad areas of use. It is not possible to balance alternate risks, provide multiple options, or targeted mitigation strategies. The problems associated with this approach have been described by Boobis et al. (2016).

Level 2 uses a discontinuous variable weight of evidence (WOE) approach to consider severity and potency. In some cases, such as dermal and eye irritation, the dose is fixed and the severity of the response is graded to determine the compartment within the class that is appropriate for the chemical. In other cases, such as acute lethality, the end point is fixed and the compartmentalization is decided on the potency. Specific target organ toxicity is an example of a level 2 scheme within CLP (ECHA 2017) that uses a combination of severity and potency to determine which compartment the chemical fits within. First, the chemical must show ‘significant’ or ‘severe’ toxicity. In this context, ‘significant’ apparently means “changes which clearly indicate functional disturbance or morphological changes which are toxicologically relevant. ‘Severe’ effects are generally more profound or serious than ‘significant’ effects and are of a considerably adverse nature which significantly impact health”. Then, the point of departure is used to assign the chemical to one of three categories. This enables a greater range of risk management options than with a level 1 scheme, because the compartments are based on severity and/or potency and the use of the same principles for developing a level 2 scheme for CMR will be explored in this paper.

Level 3 uses a continuous variable approach to incorporate a combination of severity and potency to derive doses which are of no or low concern. Potency is considered by examining the dose–response curve and determining the dose at which no or minimal effect is expected (point of departure) to which a safety/uncertainty factor is applied (Dorne et al. 2005; Dourson and Stara 1983; Dourson and Felter 1996) or by other means such as low-dose extrapolation to a low probability of effect. Severity, at times, drives the size of the safety factor that is considered necessary to ensure safety. This may be done by applying an additional factor to the point of departure if the effect is deemed to be severe (e.g., developmental abnormalities), although this does not apply in all regulatory frameworks that consider adequacy of available data and not severity of a particular effect (Dourson and Felter 1996). Each chemical is considered on the basis of the available toxicological and exposure evidence, and risk management options can be specifically designed for the situation.

To summarise:

- Codification Level 1 attempts to divide chemicals into the dichotomous bands of hazardous and non-hazardous. It does not allow for degree of hazard.
- Codification Level 2 divides chemicals into a number of bands to accommodate different degrees of hazard based on severity of effects and/or potency of the agent.
- Codification Level 3 places each chemical on a continuum of degree of hazard based on severity and/or potency.

Based on the codification level scheme we have developed, it can be seen that the GHS/CLP classification system uses both Level 1 and Level 2 hazard codification. Level 2 codification is used for acute lethality, dermal and eye irritation/corrosivity, dermal sensitization, and single exposure and repeat exposure-specific target organ toxicity. Level 1 codification is used for mutagenicity, carcinogenicity, developmental, and reproductive toxicity. Level 1 codification has also been introduced in the EU not only for a specific effect but also for a mode of action, endocrine disruption (EU 2018a, b, 2020b). In addition, it has been suggested that Level 1 coding should be expanded to immunotoxicity and neurotoxicity (EU 2020a). The rationale for expanding the use of Level 1 codification processes has not been scientifically justified, and therefore, it would not be appropriate to use them given their inherent limitations.

Leaving aside political concerns, the only scientific justification for Level 1 codification of hazard is if it is not possible to identify exposure levels that would not cause some harm. In this situation, all chemicals that have the potential to express the hazard would be considered equally hazardous and treated as if they have similar severity and similar potency. All such chemicals would, therefore, be subjected to the same risk management procedures. We have examined the schemes where Level 1 codification is used to determine whether this assumption is justifiable or whether there is a range of hazard that could reasonably be based on severity, potency, and mode of action which would mean that the assumption is not appropriate.

## Carcinogenicity

There is a wide range of potency for the induction of tumours in long-term rodent bioassays; EU CLP (ECHA 2017) guidelines state: “Experimental studies have revealed large variations in the doses of various carcinogenic substances needed to induce tumours in animals. Thus, the amounts of a chemical carcinogens required to induce tumours vary with a factor of up to 10^9^ for different compounds. It is reasonable to assume that there is similar variation in the potency of substances carcinogenic to humans (Sanner and Dybing 2005)”. Clearly, there is a continuum of potency and it is inappropriate to ignore this in hazard codification. The question of whether there is a range of severity in cancer is less easy to address. In most areas of toxicity, there is an increase in both the incidence and the severity of adverse effects with increasing dose. In long-term rodent bioassays, the number of neoplasms in the treated groups is compared with the control group. Although there is often a progression of histopathological observations such as hyperplasia leading to neoplasia, and there may be debate about where the dividing line between the two should be placed, once a neoplasm has been observed it is added to the tally. Currently, no distinction in hazard for chemicals (non-pharmaceuticals) is made between benign and malignant tumours. There is certainly a difference in mode of action, and for most carcinogenic effects, there is an obligatory progression from reversible toxicity to neoplasia, so that that health protective risk management can be based on minimizing the precursor toxic effects.

At first glance, it appears that the GHS/EU CLP and the IARC classification systems employ Level 2 hazard codification. GHS/EU CLP has Category 1 and Category 2. IARC has Groups 1–3 (previously 4). However, inspection of the schemes reveals that the category or group relates to the strength of evidence that the chemical is or is not a carcinogen and does not take into account its severity (magnitude of tumour response) or potency (dose needed to initiate and promote carcinogenicity) or mode of action.

In fact, IARC’s grouping is based on the strength of evidence as to whether the hazard is possible not on degree of hazard (IARC 2019):

Group 1: the agent is carcinogenic to humans.

Group 2A: the agent is probably carcinogenic to humans.

Group 2B: the agent is possibly carcinogenic to humans.

Group 3: the agent is not classifiable as to its carcinogenicity to humans.

The assessment is at Level 1, and this has been confirmed in the change to the name of the IARC monograph programme in 2019 when ‘Evaluation of Carcinogenic Risks’ became ‘Identification of Carcinogenic Hazards’. IARC emphasises the point that they are operating Level 1 hazard codification: “The categories of the classification refer to the strength of the evidence that an exposure is carcinogenic and not to the risk of cancer from particular exposures. The terms ‘probably carcinogenic’ and ‘possibly carcinogenic’ have no quantitative significance and are used as descriptors of different strengths of evidence of carcinogenicity in humans; ‘probably carcinogenic’ signifies a greater strength of evidence than ‘possibly carcinogenic’.”

It is not as immediately apparent that the EU/GHS CLP is a Level 1 scheme. Category 1 is for chemicals known (1A from human evidence) or presumed (1B from animal evidence) to have carcinogenic potential for humans based on the strength of evidence. Chemicals are placed in Category 2 on similar evidence “but which is not sufficiently convincing to place the substance in Category 1A or 1B”. This implies that the hazard codification is at Level 1, binary carcinogen/non-carcinogen. The different categories do not reflect the degree of hazard (severity/potency), but the strength of evidence that the hazard is possible thus making it difficult to link risk management choices to the categorisation.

The guidance in the EU (2017) seems to be less certain. The commentary seems to mix up strength of evidence with weight of evidence in terms of severity and potency. For instance, in referring to evidence from 2-year bioassays the guidance says: “In general, chemicals are evaluated for carcinogenic potential in two-year bioassays conducted in mice and rats. The chemicals produce a spectrum of responses ranging from no effects in either species to induction of malignant neoplasms in multiple tissues in both species. Between these two extremes, there are variable responses in tissues, sexes, and species which demonstrate that there are important differences among the carcinogens, as well as between the species in which they are tested. The tumour profile observed with a substance should be taken into account when considering the most appropriate classification. Evidence shows that substances which cause tumours in either multiple sites and/or multiple species *tend to be more potent carcinogens* (emphasis added) than those causing tumours at only one site in one species (Dybing et al. 1997). This is often true for substances which are mutagenic. Also, where human carcinogens have been tested in two or more species, the majority have caused cancer in several species (Tennant 1993). Thus, *if a substance causes tumours at multiple sites and/or in more than one species then this usually provides strong evidence of carcinogenicity* (emphasis added). Typically, such a tumour profile would lead to a classification in category 1B”.

This may imply that Category 1 is for ‘more potent carcinogens’ and by default Category 2 is for ‘less potent carcinogens’. The concept that Category 2 should be for ‘less potent carcinogens’ is also indirectly implied in the guidance for consideration of malignant and benign tumours: “In general, if a substance involves a treatment related increase in tumours then it will meet the criteria for classification as a carcinogen. If the substance has been shown to cause malignant tumours this will usually constitute sufficient evidence of carcinogenicity supporting Category 1B. The induction of only benign tumours usually provides a lower strength of evidence for carcinogenicity than the induction of malignant tumours and will usually support Category 2”.

This clearly shows the problem of level 1 hazard codification which cannot accommodate the ‘important differences among the carcinogens’ which is stated in the EU CLP Guidance*.* The guidance also makes the point that “in stochastic phenomena the incidence but not severity increases with dose, whereas in threshold toxicity both incidence and severity usually increase with dose”. The induction of cancer can be both stochastic and threshold depending on the mode of action (US EPA 2005 Cancer Guidelines; Wolf et al. 2019). As such, both severity and incidence can increase with dose, and for some carcinogens, it is possible to identify exposure levels below which there is no toxicological concern. This underlines that Level 1 hazard codification is not scientifically justifiable nor appropriate for use to address carcinogenicity potential.

The EU (ECHA 2017) acknowledges the problem created by the Level 1 codification for carcinogenicity in the way limits are created for the presence of categorised chemicals in commercial preparations. The guidance contains provisions for what are called Specific Concentration Limits; these set out the maximum concentration of a substance which can be used in a product based on its classification. The more severe the category, the lower the Specific Concentration Limits. This works for Level 2 hazard codifications which take into account severity and potency. Lower limits are set for the more potent substances. However, Level 1 codification hazard categories cause problems, because the potency is not reflected in the category.

This was addressed by an expert working group (EC 1999) which pointed out the following: “However, the general classification system for carcinogens does not take into account the wide range of carcinogenic potency that can be observed both in human epidemiological studies and in animal experiments. As well as the need for a system to reflect this wide range of carcinogen potencies, there are examples of carcinogens where the question of potency as such is of particular concern.”

“In some cases, it is the high potency of the substances such as dimethylsulfate and hexylmethylphosphoramide or impurities such as TCDD and certain nitrosamines which gives rise to concern and it is possible that a general limit of 0.1% does not adequately express the hazard. In other cases, substances may be classified as carcinogens although relatively high doses are needed to induce tumours. In such cases the general limit may not adequately express the hazard of a preparation containing such substances, this time by over-estimating the carcinogenicity of the preparation. Other intrinsic properties of chemical carcinogens may contribute to overall concern, such as the induction of tumours after a very short latency period, or the occurrence of tumours in many different tissue sites. This may be addressed by assigning lower specific concentration limits for such substances, if not addressed directly by the classification.”

The expert working group (EC 1999) pointed out the limitations of the Level 1 hazard codification system and went about developing a Level 2 hazard codification scheme. They reviewed the measures of potency which have been used and decided on the T25 value as it can be calculated in most cases. They used it to place substances classified as a carcinogen into ranges that define potency.

- Carcinogens of high potency: T25 value < 1 mg/kg bodyweight/day.
- Carcinogens of medium potency: 1 mg/kg bodyweight/day < T25 value < 100 mg/kg bodyweight/day.
- Carcinogens of low potency: T25 value > 100 mg/kg bodyweight/day.

The leading principle used for deriving the 1 and 100 mg/kg bodyweight/day limits was the distribution of values in the database from Gold et al. (1989) with the majority of carcinogens falling into the medium potency range.

The expert group then went on to define modifying factors that could affect the level of concern including the dose–response curve, the site/species/sex incidence of tumours, mode of action including genotoxicity, and human relevance. They describe how these factors can be used to modify the initial categorisation based on potency. For instance, a genotoxic mode of action would indicate movement to a higher potency group. Lack of human relevance would indicate movement to a lower potency group. Overall, they described a process to assign chemicals that have been shown to cause cancer to one of three categories based on potency and severity; in other words, they describe a Level 2 hazard codification scheme. At present, the Level 2 hazard codification scheme is only used to set Specific Concentration Limits, but it represents a ready-made solution to the problems caused by the Level 1 hazard codification GHS/EU classification scheme.

## Reproductive and developmental toxicity

It is appropriate to consider reproductive toxicity (effects on the fertility and sexual function) and developmental toxicity (effects on the developing offspring) separately from each other. There is a range of severity of effect on reproductive toxicity from reversible small changes in gonadal histopathology and fertility to an irreversible and complete absence of fertility. Some effects identified in the reproductive system from a standard OECD test guideline study, while measurable, have no impact on fertility and fecundity and thus have questionable relevance for hazard identification. The EU commissioned a study to determine the relative potency of substances in causing reproductive toxicity and to recommend ways of assessing potency (Muller et al. 2012). They created databases for substances that are classified for reproductive toxicity in the EU for effects on reproduction and fertility and for developmental toxicity. The potency range of substances in the database was a factor of approximately six orders of magnitude, similar to the range of potency for carcinogenicity (Sanner and Dybing 2005). Adverse effects on fertility and sexual function of a substance can differ between dose levels from small changes in testes histopathology through effects on fertility to an irreversible and complete absence of fertility. Reduced fertility or irreversible changes in reproductive organs would be considered of high concern, whereas minor, reversible changes in histopathology or weight of reproductive organ would be considered of low concern.

There is clearly a continuum of severity and potency for reproductive toxicity, indicating that there is a wide variation in the degrees of hazard in chemicals which have the potential to cause reproductive toxicity.

Developmental toxicity studies categorise adverse effects that are irreversible, such as malformations and abnormalities, to be more severe, while a lower level of concern is given to variations and retardations such as delayed ossification, wavy ribs, and other minor skeletal variations (Carney and Kimmel 2007; Chernoff et al. 1991; Rogers et al. 2004). Similarly, minor delays in growth rate or developmental landmarks would be considered of low concern, whereas reduced survival would be considered of high level. Functional changes, where assessed, which are not reversed during development would be considered more severe than functional changes which are observed early in post-natal development but later reverse or have no impact on the life of the animal. Muller et al. (2012) found that the range of potency values for developmental toxicity, like reproductive toxicity, also covered six orders of magnitude.

Piersma et al. 2011 examined all the endpoints assessed in reproductive and developmental toxicity and found that they have thresholds of adversity, therefore providing evidence for the presence of dose levels with no measurable adverse effects. It is clearly inappropriate to apply Level 1 hazard codification to reproductive and developmental toxicity as there are different levels of severity, different modes of action, and a large range of potencies and thresholds below which there are no adverse effects. Reproductive and developmental toxicity should be codified at Level 2 or 3 as the situation requires.

The Level 1 codification scheme for reproductive and developmental toxicity causes the same problems in setting Specific Concentration Limits as were described for carcinogenicity. Again, the EU commissioned a review of these difficulties and a similar approach was taken. On the basis of a review by Muller et al. (2012), a potency estimate of ED10 (dose causing an increase of 10% over control values) was used to derive three potency groups:

- High potency group ED10 value ≤ 4 mg/kg bw/day
- Medium potency group 4 mg/kg bw/day < ED10 value < 400 mg/kg bw/day
- Low potency group ED10 value 400 mg/kg bw/day.

Once the potency group has been determined, the data on the chemical are reviewed for other factors that may modify the initial potency determination. The modifying factors include the type of effect and its severity, dose–response relationship, and mode or mechanism of action. In this way, the chemical is assigned to one of three groups based on potency and severity, and in other words, this is a Level 2 hazard codification scheme within the EU CLP guidance. At present, this is used only to set Specific Concentration Limits, but it could also be applied to the overall categorisation process.

## Mutagenicity

Classification for mutagenicity within the EU/GHS CLP (ECHA 2017) scheme is for germ cell mutagenicity, although this is sometimes overlooked or misunderstood. However, within the scheme, classification as a germ cell mutagen has implications for potential carcinogenicity classification, as all germ cell mutagens are presumed to also be somatic cell mutagens (ECHA 2017). Although the concept is being challenged, mutagenicity has been assumed to be a quantal effect, present or not present, with a linear dose response with no threshold. The EU/GHS CLP (ECHA 2017) classification system reflects this with Level 1 codification with two categories based on the WOE of the chemical being able to induce heritable mutations in germ cells. Category 1A is reserved for chemicals for which there is evidence from humans, and category 1B is based on evidence from in vivo assays based on germ cell mutation. Mutations in somatic cell in vivo assays are given less weight and point towards Category 2, with the assumption that the capability to cause mutations in somatic cells is evidence that mutations could be caused in germ cells. Dose responses in germ cell and somatic cell assays are not taken into account in the weight of evidence evaluation. Indeed, they are specifically discounted, as substances that are incapable of causing heritable mutations, because they cannot reach the germ cells due to physicochemical or pharmacokinetic factors with positive results in vitro, supported by at least one positive local in vivo, somatic cell test for mutagenicity, are classified as Category 2, “Substances which cause concern for humans owing to the possibility that they may induce heritable mutations in the germ cells of humans”. Category 1A and 1B receive the hazard statement ‘May cause genetic defects’ and Category 2 receives the hazard statement ‘Suspected of causing genetic defects’. Genetic defects comprise heritable genetic damage as well as somatic cell mutagenicity.

The GHS process is clearly a Level 1 hazard codification, with all chemicals considered to have equal concerns over severity and potency. The no-threshold assumption for genotoxicity has been challenged for many years, with emphasis being placed on the need to consider the mode of action; for instance, if genotoxic damage results from damage to proteins involved in cell division, like tubulin, there is a threshold dose for such genotoxic effects (Zito 2001). Wills et al. (2015, 2016) investigated whether there were differences in potency from the results of in vitro micronucleus assays and in vivo genotoxicity assays including the rat Pig-a assay and the induction of lacZ transgene mutations in Muta™Mouse. They noted that genotoxicity tests have traditionally been used only for hazard identification, with qualitative dichotomous groupings being used to identify compounds that have the capacity to induce mutations and/or cytogenetic alterations (i.e., Level 1 hazard codification). Wills et al. were interested in determining whether they could use estimates of potency using dose–response data to derive point of departure metrics that could be used to establish human exposure limits or margins of exposure (MOEs), thereby supporting human health risk assessments and regulatory decisions. They concluded that the results of the assays could be used for more than hazard identification. More specifically, they illustrate that quantitative robust potency determinations and potency rankings can be made. Others (MacGregor et al. 2015a, 2015b; Clewell and Anderson 2016; Long et al. 2018; Metruccio and Moretto 2018) have reached similar conclusions. In addition, qualitative information on modes of action and the nature of dose–response relationships for different types of mutagenic response has revealed that there are clear, mechanistically explicable thresholds (Muller et al. 2009, Kirsch-Volders et al. 2009, Gollapudi 2017; Gollapudi et al. 2013). It is now time for serious consideration of Level 2 and 3 hazard codification schemes for mutagenicity by those classifying and risk assessing chemicals.

## Endocrine disruption

Endocrine disruption has been designated in the EU as a separate hazard. This is unique in being an MOA-based hazard categorization rather than hazard based on observation of adverse effects. A chemical can be designated to have endocrine disrupting properties if it meets the following conditions:

(a) It shows an adverse effect in an intact organism or its progeny.

(b) It has an endocrine MOA, i.e., it alters the function(s) of the endocrine system.

(c) The adverse effect is a consequence of the endocrine MOA.

All three conditions must be met, and a guidance document has been published for how to evaluate data to determine whether a chemical should be classified (EU 2018a). However, there are suggestions that classification could also be based on suspicion of endocrine disrupting activity (EU 2020b).

Classification for endocrine disruption is based on hazard identification only, severity and potency are not considered, and it is therefore a Level 1 hazard codification system. This is linked to mandated severe risk management for pesticides and biocides, and there are regulatory moves to extend it to other chemical categories. Once a biocide or pesticide has been classified as an endocrine disruptor, the chemical cannot be authorised for any use. The reason given is that scientific uncertainty remains regarding their assessment [for example as regards the existence of a safe limit of exposure (EU, 2018b)]. This contradicts EFSA’s Scientific Committee, which concluded that biological thresholds of adversity do exist and considered human and environmental risk assessment (taking into account hazard and exposure data/predictions) the best approach to inform risk management decisions in regulations that base decisions on the risk and level of concern (EU 2020b). According to EFSA’s Scientific Committee, “EDs can therefore be treated like most other substances of concern for human health and the environment, i.e. be subject to risk assessment and not only to hazard assessment”. In other words, the EFSA Scientific Committee recommended the use of a Level 2 or Level 3 hazard codification scheme for endocrine disruption. The adverse effects resulting from changes in the endocrine system fall into the categories of specific organ toxicity, carcinogenicity or reproductive toxicity. There are Level 2 hazard codification schemes available for all these adverse effects, which include consideration of MOA and dose response.

## Level 1 codification and the precautionary principle

The aim of the precautionary principle, according to an EU Parliamentary Review (EU 2017), is “to avoid causing adverse impacts in situations of scientific uncertainty”. The review contains these definitions:

Hazard: situation or risk that a substance or technology, by reason of its inherent characteristics or properties, could—under specific conditions of exposure—endanger people, property, or the environment.

Risk: likelihood of an adverse event occurring because a hazard coincides with exposure to that hazard.

Uncertainty: a situation where environmental and/or human health impacts are likely, but the probabilities are unknown, may lead to precautionary measures to reduce exposure to certain hazards. (Note from authors: the EU does not define “likely, but the probabilities are unknown”. The phrase could be taken to mean “possible” and therefore justify precautionary measures in a wide range of circumstances.)

Level 1 hazard codification schemes are applications of the precautionary principle. It is understandable that Level 1 schemes were initially adopted for carcinogenicity, mutagenicity, and reproductive toxicity in the early days of chemical safety evaluation. At that time, MOAs were unknown, and dose–response curves were unexplored with concerns over whether there were thresholds for some adverse effects. This situation met the definition of scientific uncertainty where health impacts were likely, but the probabilities were unknown and an application of the precautionary principle could be justified.

The EU Review then goes on to state that, “Where risks are established with certainty, it is the prevention principle, as enshrined in the Treaty on the Functioning of the European Union, which can be brought into play to adopt hazard prevention measures.” As the body of knowledge about the effects of chemicals increased, the MOAs and dose–response curves become defined, and the risks (when the hazard coincides with exposure) can be established and the prevention principle (taking appropriate action to prevent adverse effects) should be applied.

Endocrine disruption provides an example of the evolution of knowledge. Initially, there were concerns over the effects and the dose response being atypical as voiced by the some members of the Endocrine Society and they invoked the precautionary principle to advocate a level 1 approach (Diamanti-Kandarakis et al. 2009). The review commissioned by the EU (2018b) gathered all the evidence and concluded that biological thresholds of adversity do exist and that level 2 and 3 approaches are appropriate for endocrine disruption.

Level 2 and 3 codification schemes allow the adoption of appropriate prevention measures as required in the prevention principle. Level 1 schemes deprive policymakers of essential information necessary for decision-making. The precautionary principle could still be invoked for compounds codified using a Level 2 or Level 3 scheme, resulting in such decisions being better informed with greater scientific rigour and justification.

## Discussion

The purpose of this paper was to investigate the scientific reasons behind the controversy between hazard-based and risk-based chemical regulatory and safety assessment schemes which was highlighted by Lofsted (2011) and by the BfR (2021). Our investigation has revealed that the controversy is not between hazard and risk. It is between Level 1 hazard codification on one hand and Level 2 and 3 hazard codification on the other.

One reason that Level 1 codification creates controversy is because it divides substances into only two categories: hazardous or non-hazardous. Controversy arises, because the fewer the categories, the greater the probability of disputes and the temptation to influence the assignment of a substance to a predetermined category in either direction. The greatest probability of such problems occurs when Level 1 binary hazardous/non-hazardous hazard codification is used to trigger severe risk management decisions such as banning of chemicals for broad categories of use. The process can become a quasi-forensic exercise where evidence is brought in, in a similar way to a legal case, with each side of the binary argument amassing evidence to support its preconceived idea. A case in point is the key characteristics of carcinogens (Smith et al. 2016), which may provide useful information for the understanding of carcinogenic MOAs but which should not be used as evidence for or against a dichotomous classification. The restrictive nature of a two-choice scheme creates unnecessary angst in the process of chemical regulation.

A second reason Level 1 codification creates controversy is because it can create a false dilemma, which is defined as when only two choices are presented yet more exist, or a spectrum of possible choices exists between two extremes (LogicallyFallcious 2021). Other names for a false dilemma include all-or-nothing fallacy, either-or reasoning, black-and-white thinking, and polarization. Clearly, in the case of hazards to health posed by chemicals, more than two choices exist beyond being hazardous or non-hazardous. We know that biological response to chemicals presents a spectrum of possible choices between the extremes, and categorization schemes should reflect that reality.

Codification Level 2 hazard assessments avoid the Level 1 bifurcation and false dilemma controversies to some extent by offering a limited range of choices between the extremes and tend to reduce the built-in conflict inherent in the Level 1 process. Taking skin irritation as an example, categorising a chemical in a Level 1 process as irritating or non-irritating would hardly be helpful. However, a Level 2 process allows for multiple skin irritation categories that indicate corrosive, severe, moderate, and non-irritant, which is easily understood and properly reflects the range of possible outcomes from exposure.

Codification Level 3 assessments avoid the false dilemma completely by allowing a full range of choices between the extremes and reduces the built-in conflict even more. Level 3 assessments properly reflect the range of biological response to chemical substances when sufficient data are generated to discern the range. Data that describe the severity and potency of an adverse effect should always be used to assess the specific use of a chemical.

Level 1 schemes were adopted for carcinogenicity, mutagenicity, and reproductive toxicity in the early days of chemical safety schemes when modes of action were unknown and dose–response curves were unexplored. With greater understanding and experience, we now know that Level 1 schemes give rise to major problems and led to the development of Level 2 hazard codification schemes within the EU CLP for carcinogenicity and reproductive toxicity. These secondary schemes are only applied for setting Specific Concentration Limits and were needed to cope with problems resulting from the Level 1 codification schemes within which they sit. The studies sponsored by the EU to address this problem clearly show that the use of Level 1 hazard codification is not scientifically justified for carcinogenicity and reproductive toxicity. A pragmatic solution to the problem would be to adopt these Level 2 schemes already in the EU guidelines which take into account potency and severity for the categorisation of hazard for carcinogenicity and reproductive toxicity.

The situation is less well studied for mutagenicity, with exploration of potency and severity, and of MOAs, and their relationship to human safety still being investigated. It would be consistent with current scientific understanding of mutagenicity to determine if Level 2 or 3 hazard codification schemes could be developed.

It is difficult to see the scientific justification for the introduction of a Level 1 hazard codification scheme for endocrine disruption in the face of the evidence that, as is true for all chemicals and modes of action, there is a continuum of biological response. As such, EDs should be treated like most other substances of concern for human health and the environment with Level 3 risk assessment as reflected in an opinion from EFSA (EU 2020b) and not add to the false dilemma of a Level 1 hazard assessment.

The use of a Level 1 hazard codification scheme no longer provides value in situations where adverse effects span ranges of severity and potency when a Level 2 codification would more properly, and just as easily, categorise the range of response. Preferably, Level 3 codification processes should be used when data are available to conduct a risk assessment. There is not a major conceptual difference between Level 2 and Level 3 codification, they can both be used to select appropriate prevention measures or risk management options, although Level 3 may provide more precision and less uncertainty. The same laboratory animal-based studies are used in each of the three levels. Moving away from level 1 binary schemes would not result in the use of more animals but would make greater use of the information derived from the use of those animals.

In summary, the apparent controversy between hazard-based and risk-based chemical regulatory and safety assessment schemes is, in itself, a false dilemma—with a twist: determining human health safety does not need to rely simply on hazard OR risk, but can be properly addressed with data-specific acknowledgement of the range of adverse responses possible through the application of Level 2 or 3 codifications.

## Data Availability

Not applicable.

## Abbreviations

CLP: Classification, labelling, and packaging
ED10: Dose calculated to induce effects with an incidence or magnitude of 10% effect level above background data
EU: European Union
GHS: Global harmonisation system of classification and labelling of chemicals
IARC: International Agency for Research on Cancer
MOA: Mode of action
T25: The chronic daily dose in mg per kg bodyweight which will give 25% of the animals tumours at a specific tissue site, after correction for spontaneous incidence, within the standard life span of that species
